# Case report: Naturally occurring neurogenic stunned myocardium in a dog secondary to status epilepticus

**DOI:** 10.3389/fvets.2024.1376107

**Published:** 2024-05-10

**Authors:** Jacob Dunham, Mallory Horridge, Ji-Hey Lim, Bridget M. Lyons, Kelly Wiggen

**Affiliations:** ^1^Department of Veterinary Medicine and Surgery, University of Missouri Veterinary Health Center, Columbia, MO, United States; ^2^Cornell University Veterinary Specialists, Stamford, CT, United States

**Keywords:** ventricular dyskinesia, seizures, epileptic seizures, epilepsy, echocardiogram, esmolol, troponin

## Abstract

A 4-year-old male neutered Boston Terrier was presented with status epilepticus. He was diagnosed with idiopathic epilepsy and hospitalized with supportive care. During hospitalization, the patient developed both supraventricular and ventricular arrhythmias as well as focal left ventricular dyskinesis. Cardiac troponin I was significantly increased, which was supportive of myocardial damage. Neurogenic stunned myocardium was suspected, and the patient was treated and responded to esmolol. Follow-up echocardiography demonstrated the resolution of the ventricular dyskinesia. This report describes the clinical presentation, diagnostic findings, treatment, management, and outcome of the first reported case of naturally occurring neurogenic stunned myocardium in a dog. Electrocardiogram monitoring, cardiac troponin I, and echocardiography should be considered in patients presenting with seizure activity, especially when exhibiting cluster seizures or in status epilepticus.

## 1 Introduction

Neurogenic stunned myocardium (NSM) is defined as cardiovascular dysfunction after a neurologic event causing autonomic dysregulation (1). A variety of neurological insults, such as seizures or subarachnoid hemorrhage, have the potential to lead to significant catecholamine surges (1, 2). At high concentrations, catecholamines exhibit cardiotoxic effects and may cause infarction-like changes such as arrhythmias and ventricular wall motion abnormalities (1, 2). These changes can be life-threatening if not promptly identified and managed (2). Unfortunately, there is no official consensus for the diagnosis of NSM, thus it is considered a diagnosis of exclusion, with the primary rule out in humans being acute myocardial infarction (3). Medical management consists of the administration of beta-blockers to reduce catecholamine effects on the myocardium (2, 4). Although it has been clinically studied in human medicine (1, 2) and experimentally documented in dogs (5), NSM has not been reported as naturally occurring in the veterinary literature.

## 2 Case description

A 4-year-old male-neutered Boston Terrier was presented to the University of Missouri Veterinary Health Center in status epilepticus. The patient began exhibiting seizure activity 2 years prior to presentation, with each episode occurring approximately 6 months apart, with no known history of cluster seizures. Due to the duration between seizure episodes, anti-epileptic drug therapy was not instituted. On the day of the presentation, the dog had eight tonic clusters without returning to normal mentation between episodes (6). The dog was presented to his primary care veterinarian, where two doses of midazolam were administered intravenously (IV) (0.79 mg/kg) and one dose of diazepam orally (1.1 mg/kg). The dog was subsequently referred for further management and diagnostic testing.

Upon presentation, the patient was mentally inappropriate, dull, and had a generalized tonic–clonic seizure followed by focal motor seizures characterized by rhythmic contractions of facial muscles and chewing movement. Physical examination revealed hyperthermia (105.1°F/40.6°C) and hyperemic mucous membranes. On cardiothoracic auscultation, no murmur or arrhythmia was noted, and an electrocardiogram revealed sinus tachycardia (160 beats/min). Doppler blood pressure was recorded to be 130 mmHg. Blood gas analysis showed increased hematocrit (60%, reference range 38–50), hypernatremia (151.3 mmol/L, reference range 136–142 mmol/L), hypokalemia (3.30 mmol/L, reference range: 3.8–5 mmol/L), hyperchloremia (114.3 mmol/L, reference range: 95–103 mmol/L), ionized hypermagnesemia (1.04 mmol/L, reference range: 0.4–0.65 mmol/L), hyperglycemia (155 mg/dL, reference range: 70–110 mg/dL), hyperlactatemia (3.8 mmol/L, reference range: 0.6–2.2 mmol/L), increased blood urea nitrogen (27 mg/dL, reference range: 7–15 mg/dL), and elevated creatinine (1.6 mg/dL, reference range: 0.6–1.3 mg/dL). The dog was administered a phenobarbital loading dose (16 mg/kg) IV, two doses of midazolam (0.5 mg/kg) IV, and placed on a midazolam constant rate of infusion (CRI) (0.2 mg/kg/h) overnight with the maintenance fluid therapy at 40 mL/kg/day of isotonic fluids (PlasmaLyte A).

The following morning, magnetic resonance imaging revealed mild hyperintensity within the piriform lobe bilaterally on T2-weighted and FLAIR sequences without any evidence of contrast enhancement. These lesions were consistent with postictal changes. Cerebrospinal fluid analysis was unremarkable. Thus, the dog was diagnosed with idiopathic epilepsy, and phenobarbital was continued (2.5 mg/kg IV q12h) for maintenance. The midazolam CRI was discontinued at that time.

**Figure 1:**
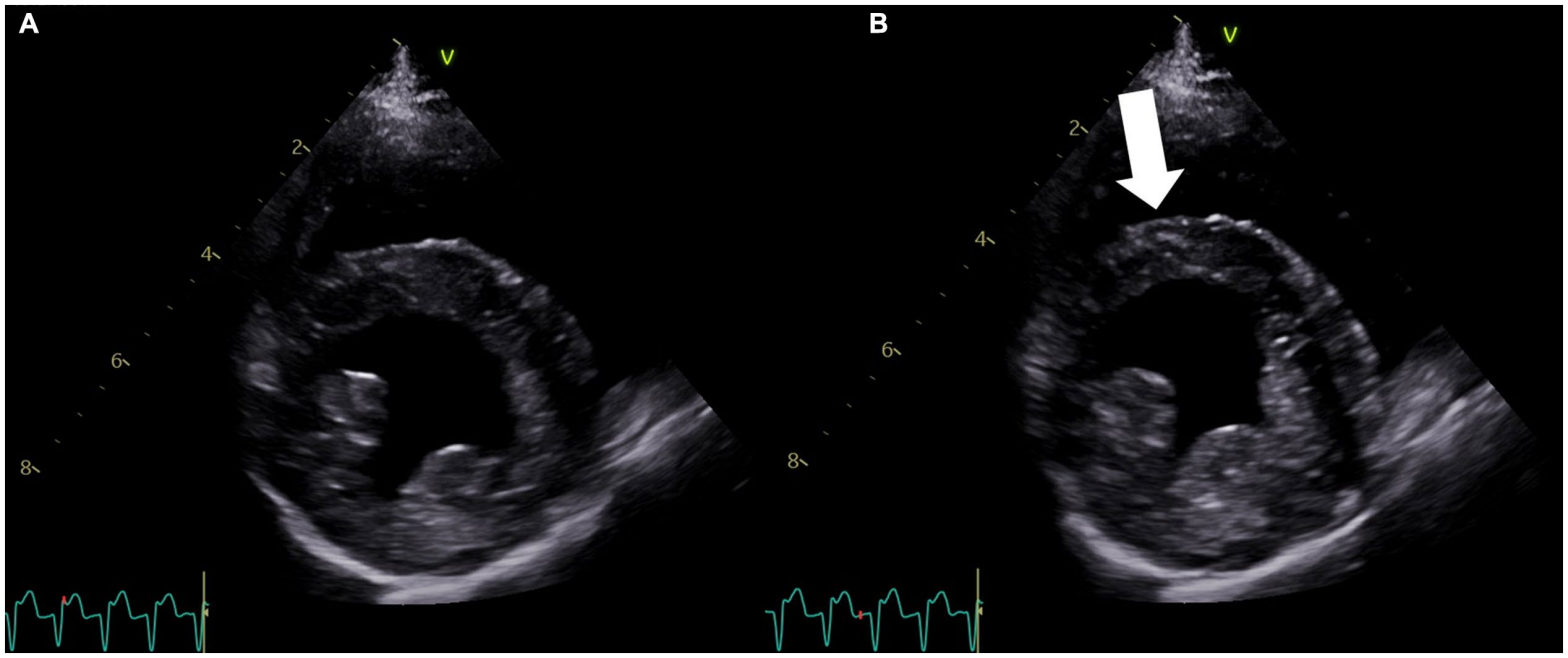
Right parasternal four-chamber short-axis echocardiogram image at the level of the left ventricle obtained on the second day of hospitalization. **(A)** View of the left ventricle in diastole. **(B)** View of the left ventricle during systole showing the dyskinetic motion of the interventricular septum (arrow). The concurrent lead II electrocardiogram shows ventricular tachycardia in both **(A,B)**.

**Figure 2:**
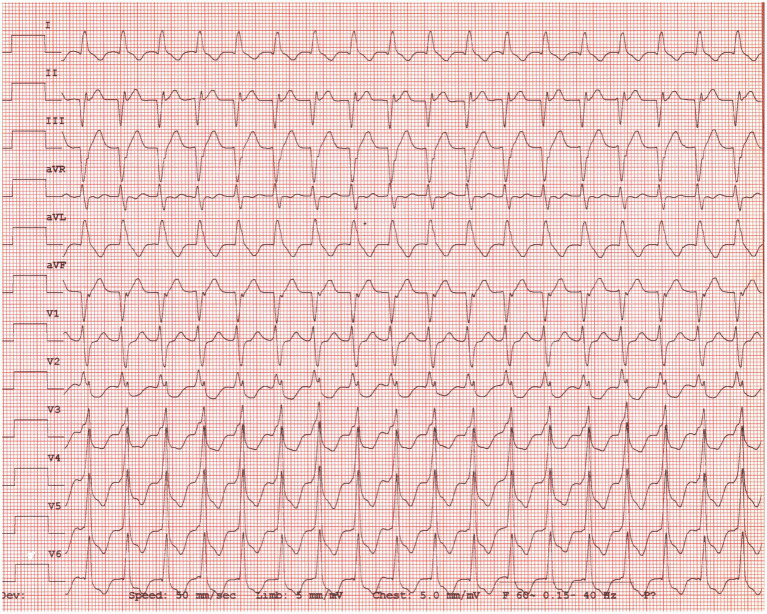
Ten-lead electrocardiogram consistent with ventricular tachycardia. Paper speed = 50 mm/s and amplitude = 10 mm/mV.

Later that night, the patient became hyperthermic (105.9°F/41.1°C). Ampicillin-sulbactam (30 mg/kg IV q8h) was prescribed due to concern for silent regurgitation and potential aspiration pneumonia. Initially, the patient’s temperature decreased, but it subsequently increased to 106°F (41.1°C). At this time, the heart rate had increased to 186 beats per minute, and facial focal epileptic seizures were noted. Levetiracetam (60 mg/kg IV) and midazolam (0.3 mg/kg IV) were administered. A lead II electrocardiogram was suspicious for ventricular tachycardia. Lidocaine (2 mg/kg IV, repeated twice) was given immediately followed by a lidocaine CRI at 30 mcg/kg/min, which was progressively increased over 3 h to 100 mcg/kg/min due to persistent ventricular arrhythmias. As the CRI rate increased, a total of eight additional lidocaine boluses (2 mg/kg each) were administered. Due to the lack of response, lidocaine was discontinued, and an amiodarone CRI (0.8 mg/kg/h) was started with minimal response. An echocardiogram identified focal dyskinesis (an outward motion of the wall during systole) of the inferoseptal and anteroseptal portions of the interventricular septum (Figure 1; Supplementary Video S1). Cardiac troponin I was markedly elevated (28.48 ng/mL, reference range: 0.00–0.05 ng/mL). A 10-lead electrocardiogram was performed and confirmed persistent ventricular tachycardia (Figure 2, heart rate 260 beats/min) with intermittent periods of accelerated idioventricular rhythm. Due to the presence of ventricular arrhythmias, focal dyskinesis, and a marked elevation in cardiac troponin I, NSM was suspected. Due to the poor response to amiodarone, it was discontinued, and two boluses of esmolol (50 mcg/kg/dose IV) were administered. During the second bolus, the patient was converted into a normal sinus rhythm with intermittent single ventricular premature complexes. An esmolol CRI was started and titrated to effect (10–20 mcg/kg/min). Dexamethasone (0.14 mg/kg IV q24h) was administered, and levetiracetam (30 mg/kg IV q8h) was continued.

On the fourth day of hospitalization, the patient developed a superficial corneal ulcer in the right eye, and a triple antibiotic ophthalmic ointment was prescribed. The patient was able to maintain sternal recumbency but was unable to ambulate. The patient started eating, had improved mentation, and was transitioned to oral medications: atenolol (initially 0.5 mg/kg *Per Os* (PO), then increased to 0.75 mg/kg PO q12h), prednisone (0.8 mg/kg PO q24h), amoxicillin-clavulanic acid (14.8 mg/kg PO q12h), and phenobarbital (2.5 mg/kg PO q12h).

**Figure 3:**
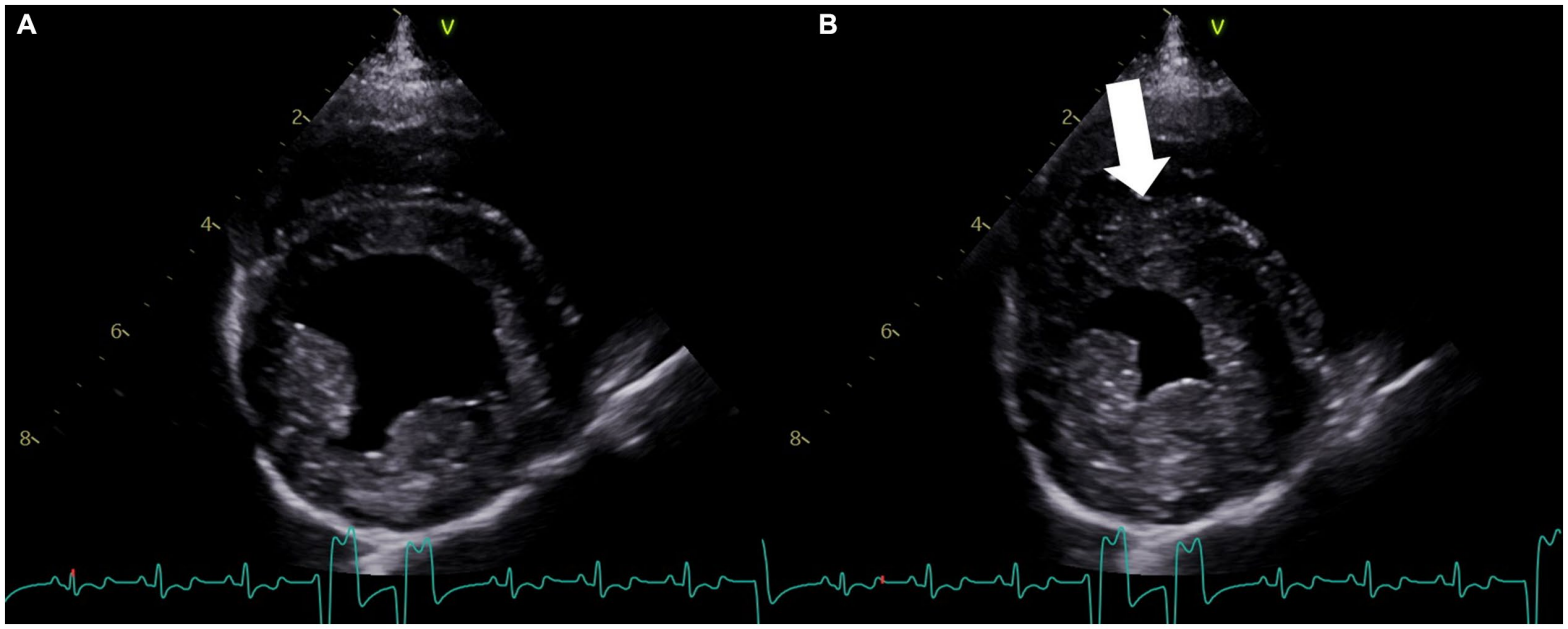
Right parasternal four-chamber short-axis echocardiogram image at the level of the left ventricle obtained on the fifth day of hospitalization. **(A)** View of the left ventricle in diastole. **(B)** View of the left ventricle during systole, showing resolution of the previously noted dyskinesis and residual hypokinesis (arrow). The concurrent lead II electrocardiogram shows an underlying sinus rhythm with intermittent accelerated idioventricular rhythm in both **(A,B)**.

On the fifth day of hospitalization, a repeat echocardiogram revealed improvement in ventricular motion, with the resolution of the dyskinesis and residual hypokinesis (Figure 3; Supplementary Video S2). Cardiac troponin I was significantly improved (12.41 ng/mL). The patient was transitioned to oral extended-release levetiracetam (40 mg/kg PO q12h).

On the sixth day of hospitalization, the patient developed severe skin lesions under the adhesive telemetry pads as well as urine scald. Silver sulfadiazine cream was applied to the affected areas, and an indwelling urinary catheter was placed. Recurrent paroxysmal ventricular tachycardia occurred and responded well to a bolus of lidocaine (2 mg/kg IV). Oral mexiletine (4.2 mg/kg PO q8h) was prescribed, and the atenolol dose was increased to 0.94 mg/kg PO q12h.

On the seventh day of hospitalization, the patient began regurgitating, which prompted the administration of maropitant citrate (1 mg/kg IV q24 h) and metoclopramide (2 mg/kg/day). New dermal reactions were noted due to the telemetry pads, and telemetry was discontinued. When assisted to stand, the patient was weakly ambulatory and tetraparetic.

The patient was discharged from the hospital on the eighth day of hospitalization. The following were the medications included: atenolol (0.94 mg/kg PO q12h), phenobarbital (2.5 mg/kg PO q12h), levetiracetam extended-release tablets (41.7 mg/kg PO q12h), mexiletine (4.2 mg/kg PO q8h), the triple antibiotic ophthalmic solution applied to the right eye q8h, a tapering course of prednisone (0.8 mg/kg PO q24h for 4 days, then decreased to 0.4 mg/kg PO q24h, then decreased to 0.4 mg/kg PO q48h for 4 days, and then discontinued), maropitant citrate (2 mg/kg PO q24h), and diphenhydramine (2 mg/kg PO q8h). Midazolam was also dispensed, with instructions to be administered intranasally at home if the patient had an epileptic seizure (0.5 mg/kg).

Over the next several days, the patient began to decline at home with persistently dull mentation, excessive hypersalivation, tenesmus, and hyporexia to anorexia. Due to the declining quality of life, the owners elected euthanasia and declined necropsy.

## 3 Discussion

In humans, NSM can occur following neurological events, such as seizures or subarachnoid hemorrhage, leading to cardiac damage and dysfunction (1). This phenomenon has been well-documented in human medicine (2) but has only been described in a single experimental canine model (5). To the authors’ knowledge, this is the first case report of naturally occurring NSM in a dog.

In humans, NSM is postulated to be due to a rapid increase in catecholamine release after regions of the brain that control the autonomic nervous system have been damaged (2). Catecholamines, such as epinephrine and norepinephrine, bind to beta-receptors on the surface of cardiac myocytes, activating a signaling cascade that ultimately leads to an increase in contractility, heart rate, and conduction speed, all of which can increase myocardial oxygen consumption (7). When neurological insults occur, such as cluster seizures or traumatic brain injury, it can lead to excessive stimulation of the sympathetic nervous system and an increased production of circulating catecholamines (1). The primary site for the synthesis of norepinephrine, the locus coeruleus, is in the posterior region of the rostral pons and can be stimulated during neurologic activity or injury to induce excessive catecholamine production (1, 8). This catecholamine surge can be cardiotoxic due to excessive activation of the beta-adrenergic receptors on the myocardium, causing calcium overload, depletion of adenosine triphosphate, and subsequently cell death (1). Evidence of myocardial dysfunction can be identified with elevated serum troponin levels, left ventricular dysfunction, and arrhythmias (1). A diagnosis of NSM may be missed if clinicians are unaware of this potential sequela, given the absence of reports in clinical veterinary medicine. In the last two decades, NSM has been reported more frequently in human medicine (9). This may be due to increased awareness and monitoring in patients with status epilepticus, traumatic brain injury, or subarachnoid hemorrhage rather than increased occurrence (9). Failure to promptly identify and address these secondary cardiovascular effects can have life-threatening consequences such as fatal arrhythmias, pulmonary edema, and increased intubation time (10).

In human medicine, echocardiography is used to diagnose and monitor left ventricular wall dysfunction in patients with neurologic insults and who are at risk for neurogenic cardiomyopathies (2, 10–14). New myocardial dysfunction, characterized by global or regional wall abnormalities, such as hypokinesis or dyskinesis, is the primary criterion for the diagnosis of NSM (4). The patient described in this case study presented with status epilepticus, likely the inciting event for the development of NSM, and later developed arrhythmias. Electrocardiographic abnormalities are also common in patients with NSM (4), including in experimental dog models (15), and the arrhythmias in the dog in this report spurred the recommendation for echocardiography. The echocardiogram in this case confirmed the presence of a regional wall abnormality (dyskinesis, outward bulging of the ventricular myocardium during systole; Figure 2; Supplementary Video S1).

There are three main mechanisms that cause cardiac injury in patients with NSM: coronary vasospasm secondary to increased circulating catecholamines, ischemia due to high oxygen demand, and direct myocardial injury by catecholamines (4). Unfortunately, the clinical signs of NSM are similar to those of myocardial infarction, making it difficult to obtain a definitive diagnosis of NSM based on clinical presentation alone (1, 16). Additional diagnostic testing, such as coronary angiography (1, 2), is needed to rule out myocardial infarction. In contrast to myocardial infarction, patients with NSM neither have preexisting heart disease or evidence of atherosclerosis nor have evidence of myocardial necrosis surrounding the affected coronary arteries on histopathology (1).

Bulsara et al. (17) created criteria to aid in distinguishing patients with stunned myocardium due to subarachnoid hemorrhage from myocardial infarction, including no known history of cardiac disease, new onset of cardiac dysfunction without impaired coronary circulation, wall motion abnormalities that do not correspond to ischemic changes on electrocardiography, and cardiac troponin values less than 2.8 ng/mL in patients with ejection fraction less than 40%. Further distinctions can be made using angiography, as coronary artery stenosis is commonly found with acute myocardial infarction but not with NSM (10, 14). In veterinary medicine, myocardial infarctions and coronary stenosis are rarely documented but ideally should be ruled out before making the diagnosis of NSM (18). Nevertheless, while coronary angiography is important for the evaluation of coronary artery disease, this diagnostic is not currently recommended in humans if NSM is suspected (1).

In humans, a diagnosis of NSM carries a poor prognosis if left untreated, as uncontrolled cardiac dysfunction can be life-threatening. Beta-blockers, such as esmolol, are generally the drug class of choice for the treatment of NSM in humans and have been shown to improve clinical signs, cardiac function, and prognosis, though specific treatment guidelines have not been developed (2, 3, 19, 20). A case report by Papadis et al. (21) documented a human patient diagnosed with Takotsubo cardiomyopathy, a similar and often conflated neurological cardiomyopathy with a similar pathophysiology as NSM, which occurs secondary to intense emotions or stress. With Takotsubo cardiomyopathy, there is an abrupt increase in catecholamine release or catecholamine “storm” due to emotional stress; similarly, with NSM, there is an abrupt increase in catecholamine release due to neurologic injury. The person in the aforementioned report was successfully treated using beta-blockers with complete recovery of left ventricular function. In different studies on both Takotsubo cardiomyopathy and subarachnoid hemorrhage, esmolol administration resulted in an improvement of ventricular wall dysfunction and a reduction of cardiac troponin I levels, with the majority achieving complete recovery (2, 14, 16, 17).

In this case study, the patient was diagnosed with epileptic seizures and had no prior history of cardiac disease. The dog presented with status epilepticus, which the authors suspect was the inciting central nervous system trigger for excessive catecholamine release. During hospitalization, the patient developed acute, severe ventricular arrhythmias that were refractory to lidocaine and amiodarone, which prompted a cardiology consultation. The echocardiogram revealed focal left ventricular dysfunction, ventricular arrhythmias (accelerated idioventricular rhythm and ventricular tachycardia), and elevated cardiac troponin I levels. The patient was prescribed a beta-blocker following these diagnostics, which resulted in significant improvement in the patient’s arrhythmias. While there is no literature describing the use of beta-blockers in veterinary neurogenic cardiomyopathy cases, the authors elected to use esmolol, extrapolating from human medicine (2, 8, 19, 20). The patient’s response to esmolol, coupled with the echocardiogram findings, increased the authors’ suspicion of NSM. A repeat echocardiogram performed after 48 h revealed that the affected segment showed significant improvement in motion, with residual hypokinesis present by that time (Figure 3; Supplementary Video S2). As the patient had no prior history of cardiac disease or evidence of primary diseases that could predispose to ischemic events, myocardial infarction was considered less likely, although angiography was not performed to assess the coronary vasculature. It is also important to note that myocardial infarctions are rare in dogs and typically result from disease processes causing concentric hypertrophy or a hypercoagulable state (18).

Given the patient’s history of idiopathic epilepsy and status epilepticus, new-onset arrhythmias, increased cardiac troponin I, reversible cardiac dysfunction, and response to beta-blocker therapy, the authors believe there is evidence to support this study as the first case of naturally occurring NSM in a dog. Electrocardiography, cardiac troponin I, and echocardiography should be considered in patients presenting with seizure activity, especially when exhibiting cluster seizures or status epilepticus.

## Data availability statement

The original contributions presented in the study are included in the article/Supplementary material, further inquiries can be directed to the corresponding author.

## Ethics statement

Ethical approval was not required for the studies involving animals in accordance with the local legislation and institutional requirements because this is a case report, not a clinical trial. Written informed consent was obtained from the owners for the participation of their animals in this study.

## Author contributions

JD: Writing – original draft, Writing – review & editing. MH: Writing – original draft, Writing – review & editing. J-HL: Writing – original draft, Writing – review & editing. BL: Writing – original draft, Writing – review & editing. KW: Writing – original draft, Writing – review & editing.

## Supplementary material

The Supplementary material for this article can be found online at: https://doi.org/10.6084/m9.figshare.25033733.v1; https://doi.org/10.6084/m9.figshare.25054832.v1.

Supplementary Video S1 Right parasternal four chamber short axis echocardiogram image at the level of the left ventricle obtained on the second day of hospitalization. There is dyskinetic motion of the interventricular septum, characterized by an outward motion of the inferoseptal and anteroseptal portions. The concurrent lead II electrocardiogram shows ventricular tachycardia.

Supplementary Video S2 Right parasternal four chamber axis echocardiogram image at the level of the left ventricle obtained on the fifth day of hospitalization. There is resolution of the previously-noted dyskinesis with residual hypokinesis of the affected segment of the interventricular septum. The concurrent lead II electrocardiogram shows an underlying sinus rhythm with intermittent accelerated idioventricular rhythm.
